# Microexpressions Are Not the Best Way to Catch a Liar

**DOI:** 10.3389/fpsyg.2018.01672

**Published:** 2018-09-20

**Authors:** Judee K. Burgoon

**Affiliations:** Center for the Management of Information, University of Arizona, Tucson, AZ, United States

**Keywords:** lying, deception, deception detection, microexpression, emotion, social signals, rigidity

Microexpressions are lauded as a valid and reliable means of catching liars (see Porter and ten Brinke, 2010). However, there are many reasons to question what I will call microexpression theory (MET).

For MET to be supported, several propositions must hold true: One, deception produces internal negative emotional experiences. Two, these internal experiences have associated outward expressions, including microexpressions. Three, microexpressions are uncontrollable. Four, these expressions are reliable and valid indicators of deception. Five, microexpressions occur frequently enough to be detectable. Six, detected microexpressions successfully distinguish truth from deception. Let me address each of these propositions in turn. I will then offer an alternative theory accounting for truth versus deception indicators under multiple circumstances.

## Does deception produce negative emotional experiences?

During this discussion, I distinguish among emotional *experiences*, emotional *expressions*, and *felt emotions*. Emotional experiences are internal events, not measured directly but inferred from measurable physiological states, and labeled as emotional expressions. Felt emotions are an individual's subjective reporting of one's state. Another important distinction is between macro- [>0.5 s; see (Yan et al., 2013)] and microexpressions. Most of the research on emotions during deception examines macro displays rather than fleeting microexpressions (e.g., DePaulo et al., 2003; Warren et al., 2009; Okubo et al., 2012; ten Brinke et al., 2012). Ability to predict deception from the former does not equal ability to predict deception from the latter.

So, yes, deception can produce the prototypical negative felt emotions of guilt, shame, sadness, and fear; but it can also produce positive emotions of relief, enjoyment, pleasure, and what is called duping delight—pleasure at succeeding with one's lies (Ekman and Friesen, 1969). Additionally, prevaricators may experience arousal alone, without a felt emotion, or a blend of emotions, producing complex displays that range from non-aroused to highly aroused and unpleasant to pleasant (Burgoon et al., 1989). If stakes are low, liars may not experience any particular emotion, making them indistinguishable behaviorally from truth-tellers (Hartwig and Bond, 2011).

## Do deception-instigated emotional experiences have associated outward displays?

The Ekman Facial Action Coding System (FACS) specifies particular Action Units (AUs) of the face that are associated with each emotion. For example, fear entails seven different facial muscle movements. However, as Barrett (2006) concluded, felt emotions lack a one-to-one correspondence with emotional expressions; there are no unique and specific somatovisceral changes that correspond to specific emotional expressions. Put another way, a single *felt emotion* can give rise to multiple *expressions*, and multiple *felt emotions* can give rise to the same *expression*. For example, felt fear can be displayed as anxiety, anger, contempt, or surprise. An experiment intending to examine the coherence between subjective emotions and prototypical AUs for fear showed participants the iconic scene from the movie *The Shining* of axe-wielding Jack Nicholson about to attack his terrified wife hiding in the bathroom (Fernández-Dols et al., 1997). Of 14 participants reporting exclusively disgust and 2, fear, only 1 and 2, respectively, showed the AUs associated with those felt emotions. Of 11 reporting surprise, none displayed the prototypical display. This study offered strong demonstration that felt and expressed emotions are often not aligned. Elsewhere, both a frustrating task and a delightful one elicited smiling from participants (Hoque et al., 2012).

Only at the risk of false positives, then, can one infer backward from observed displays that the sender is lying. Put differently, deception does not reliably produce negative emotions and negative emotions do not reliably signal deception.

## Are deception-instigated microexpressions uncontrollable?

Some are. But they appear to be partial or longer than the traditional definition of a microexpression. In an investigation of countermeasures, Hurley and Frank (2011) found that liars could not completely inhibit eyebrow or lip corner movement despite instructions to do so during a mock crime interrogation. In a comprehensive analysis of 78 public pleas for the return of missing children, deceivers failed to simulate sadness and grief to the degree truthful pleaders did and they leaked displays of happiness (ten Brinke et al., 2012). These, however, were full emotional expressions lasting nearly a second or longer. The microexpressions did not differ between truth and deception. The analysis of 1,711 expressions revealed that high-intensity emotions were harder to conceal than low-intensity ones during masking. There were no cases of complete microexpressions (simultaneously present in the upper and lower face), and only 25% of the participants displayed the partial expressions (Porter et al., 2012).

Other expressions, such as the eyebrow flash or contempt, are inherently social, voluntary, and controllable (Grammer et al., 1988). As social signals, they are responsive to context. This is a central reason why microexpressions are a poor telltale sign of lying, because they can be masked, minimized, exaggerated, or neutralized, especially during deception (Ekman, 2009). Masking involves disguising felt emotions, as in replacing fear with a show of defiance. Minimizing involves suppressing the intensity of an expression. Exaggeration entails intensifying an expression, as in dialing up a display of surprise when accused of a transgression. Neutralizing entails concealing a felt emotion by eliminating its outward expression entirely.

This management of facial expressions follows what Ekman and Friesen (1969) labeled display rules. Cultures designate what emotions are appropriate, sanctioned, or punished if displayed; how such displays should appear; and the consequences of their display. This designation of social signals during deception has received considerable scholarly attention (Driskell et al., 2012). Buller and Burgoon (1994, 1997) labeled such regulation of deception expressions as strategic communication. Their argument, bolstered by numerous investigations, is that much of our nonverbal behavioral repertoire is manageable and managed.

More broadly, because facial expressions are part of a social signal system, they fulfill a variety of functions beyond simply revealing one's internal emotional reactions (e.g., Buck, 1991; Chovil, 1991; Buck et al., 1992; Barrett, 2006). During social interaction, individuals purposely regulate and withhold expressions of felt emotions, and they enact expressions of emotions they do not feel. “Emotional expressions, then, can be used purposely in deception to communicate symbolically information that has very little to do with the communicators' felt emotions” (Buller and Burgoon, 1994, p. 387).

## How reliable and valid are microexpressions as indicators of deception?

High stakes circumstances may prompt some leakage, though not necessarily of microexpressions. ten Brinke and Porter (2012) found that liars pleading for the return of their missing children displayed more upper face surprise and lower face happiness than truthful pleaders, making these expressions candidates for detecting deception. “The ‘grief' muscles (*corrugator supercilii, depressor anguli oris*) were more often contracted in the faces of genuine than deceptive pleaders. Subtle contraction of the *zygomatic major* (masking smiles) and full contraction of the *frontalis* (failed attempts to appear sad) muscles were more commonly identified in the faces of deceptive pleaders” [(ten Brinke et al., 2012), p. 411].

Contrariwise, Pentland et al. (2015) found during a Concealed Information Test (CIT) that deceptive (guilty) respondents exhibited less contempt and more intense smiles than truthful (innocent) respondents. Contempt and less intense smiling would be expected of liars, not truth-tellers. Numerous studies have found that deceivers often show appeasement or fake smiles that can be mistaken as signals of pleasure, comfort, or enjoyment. In their experiment comparing cheaters (who lied) with cooperators (who were truthful), Okubo et al. (2012) concluded that, “cheater detection based on the processing of negative facial expressions can be thwarted by a posed or fake smile, which cheaters put on with higher intensity than cooperators” (p. 217).

These patterns would result in false positives. Thus, some expressions like smiling are not uniquely associated with deception, and some emotional expressions—both micro and macro—can be associated with either truth or deception.

## Do microexpressions occur with enough regularity to be detectable?

False negatives are commonplace. In one of the very few investigations of microexpression frequency, Porter and ten Brinke (2008) coded 700 high-stakes genuine and falsified emotional expressions and found only 2% were microexpressions. Their subsequent analysis of these high-stakes pleadings found only six instances of microexpressions among deceivers and slightly more (8) among genuine pleaders (ten Brinke and Porter, 2012), obviating the role of microexpressions as sufficiently frequent or exclusive to catch deception. Legions of law enforcement personnel and airport security Behavioral Detection Officers have been trained to look for microexpressions as “telltale” signs of nefarious intent. However, testimony to the U.S. Congress revealed that only 0.6% out of 61,000 passenger referrals to law enforcement in 2011 and 2012 resulted in arrests (U.S. Government Accountability Office, 2013), and a 2017 ACLU report concluded the behavioral observation approach was based on biased, weak, and junk science (Cushing, 2017).

## Do detected microexpressions successfully distinguish truth from deception?

Recent empirical results are not encouraging. Porter et al. (2012) found that untrained observers were unable to distinguish truth from deception. In particular, they could not distinguish sadness, fear or disgust—all emotions thought to be associated with deception. Pentland et al. (2015) found during a CIT that deceivers' and truth-tellers' smiling intensity was essentially the same on target questions and only differed on neutral questions. Between-subjects classification accuracy was below 56%. These kinds of results led my colleagues and me to seek an alternative approach.

## Alternative theory: the rigidity effect

An alternative hypothesis that offers a reliable and valid set of indicators is what has been called the rigidity effect (RE). RE postulates that extemporaneous deception under high stakes leads to an initial freeze response. In interpersonal deception theory (IDT), Buller and Burgoon (1996) (Burgoon, 2014, 2015a,b; see also Burgoon et al., 2014) argued that deceivers attempt to manage their nonverbal behavior and overall image so as to appear credible (strategic communication) while simultaneously attempting to control behaviors that are detrimental to their performance (non-strategic behavior). If efforts to appear natural, expressive, and relaxed are overridden by attempts to suppress signs of discomfiture, the overcontrol will backfire.

Early research focused on gestural and postural activity. Zuckerman et al. (1981) theorized that contrary to the stereotype of liars being fidgety and nervous during an interview, liars may adopt a stiff, wooden posture due to overcontrol. Several experiments confirmed that deceivers reduced their gestural, foot, and overall kinesic animation relative to truthtellers (e.g., Buller and Aune, 1987; DePaulo et al., 2003; Caso et al., 2006; Mullin, 2012), suggestive of participants seeking to limit incriminating behaviors.

As automated measurement advanced, more investigations pursued dynamic ocular and facial displays of emotion. These, too, showed the RE pattern of depressed activity. Studies of blink rates regularly found inhibition of blinking during deception (Leal and Vrij, 2008). Hurley and Frank (2011) found that suppressing a given facial emotion during deception resulted in suppressing all facial expressions. Pentland et al. (2014, 2015, 2016) and Pentland and Zhang (2016) found deceivers reduced several emotion-relevant face and head movements.

Two experiments (Pentland et al., 2017) tested the RE directly with videotapes from high-stakes experiments in which emotional and microexpressions were captured with the Computer Expression Recognition Toolkit. In the first experiment, guilty subjects completing a CIT (which controls for cognitive load) showed far less variance in four deception-relevant emotions (disgust, fear, sadness, and surprise) than did innocent subjects when responding to target questions. In the second experiment, which measured variance in 10 facial movements, the guilty (deceivers) showed REs on 8 during presentation of target images. In a separate test (Twyman et al., 2015), rigidity persisted despite participants being asked to employ countermeasures to offset it.

## Causal mechanisms

A question that needs to be resolved is the causal mechanisms that produce the RE. Does it reflect an involuntary reflex and defensive reaction associated with flight or fight (Twyman et al., 2015), a cessation of activity due to too much demand on cognitive resources and working memory (ten Brinke and Porter, 2012; Sporer, 2016), a temporary orientation response while absorbing contextual information (Le Poire and Burgoon, 1996), or intentional behavioral control and impression management (Grazioli et al., 2006; Lee et al., 2009; Twyman et al., 2011)? Sporer (2013, 2016) and Twyman et al. (2014) nicely articulates these alternatives. If the inhibition of movement^1^ is temporary while the deceiver decides how to respond, it may better reflect an adaptive response in line with IDT that is only evident if there is sufficient time for dynamics to be observed (Duran et al., 2013). (This would make rigidity a misnomer.) As already noted, immobility also is affected by distressful emotions (e.g., fear, anxiety, shame) and grave consequences (e.g., physical pain, financial loss, imprisonment) and by opportunities and cognitive resources for preparation. Speculatively, it can be hypothesized that *degree of behavioral inhibition will be positively related to one's emotional distress and the severity of the stakes involved and inversely related to opportunity to plan and adapt responses*.

Needed are experiments that tease out these effects and identify significant moderators. Methods like the cognitive interview [(Köhnken et al., 1999; Vrij, 2015); but cf. (Levine et al., 2018)] to induce higher cognitive load may further test cognitive resources, and the Twyman et al. (2015) experiments employing various countermeasures may control and test alternative influences articulated in Burgoon (2015a,b). The increased complexity despite reduced displacement found by Duran et al. (2013) also forecasts the importance of dynamic as well as static facets of emotions.

## Summary

The time has come to look beyond fleeting, infrequent and minuscule emotional expressions to movements themselves and not to their presence but their absence. Deception produces positive as well as negative emotional experiences and sometimes no emotions at all. Felt emotions do not have a one-to-one correspondence to outward expressions, and microexpressions are especially rare, leading to false negatives and false positives. Discerning initial rigidity and temporal patterning of facial behavior may greatly increase the viability of facial movements in catching a liar.

## Author contributions

The author confirms being the sole contributor of this work and approved it for publication.

### Conflict of interest statement

The author declares that the research was conducted in the absence of any commercial or financial relationships that could be construed as a potential conflict of interest.

## Footnotes

^1^This is not to be confused with Darwin's inhibition hypothesis that deceivers fail to inhibit some emotional aspects—leakage—while also failing to adequately simulate other aspects of genuine emotional expressions.

## References

[B1] Barrett L. F. (2006). Solving the emotion paradox: categorization and the experience of emotion. Person. Soc. Psychol. Rev. 10, 20–46. 10.1207/s15327957pspr1001_2 16430327

[B2] Buck R. (1991). Social factors in facial display and communication: a reply to Chovil and others. J. Nonverbal Behav. 15, 155–162. 10.1007/BF01672217

[B3] Buck R. Losow J. I. Murphy M. M. Costanzo P. (1992). Social facilitation and inhibition of emotional expression and communication. J. Pers. Soc. Psychol. 63, 962–968. 10.1037/0022-3514.63.6.962 1460562

[B4] Buller D. B. Aune R. K. (1987). Nonverbal cues to deception among intimates, friends, and strangers. J. Nonverbal Behav. 11, 269–290. 10.1007/BF00987257

[B5] Buller D. B. Burgoon J. K. (1994). Deception: strategic and nonstrategic communication, in Strategic Interpersonal Communication, eds Daly J. A. Wiemann J. M. (Hillsdale, NJ: Erlbaum), 191–223.

[B6] Buller D. B. Burgoon J. K. (1996). Interpersonal deception theory. Commun. Theor. 6, 203–242. 10.1111/j.1468-2885.1996.tb00127.x

[B7] Buller D. B. Burgoon J. K. (1997). Emotional expression in the deception process, in Communication and Emotion, eds Andersen P. A. Guerrero L. K. (Orlando, FL: Academic Press), 381–402.

[B8] Burgoon J. K. (2014). Interpersonal deception theory, in Encyclopedia of Lying and Deception, ed Levine T. R. (Thousand Oaks, CA: Sage), 532–536.

[B9] Burgoon J. K. (2015a). Deception detection accuracy, in International Encyclopedia of Interpersonal Communication, eds Berger C. R. Roloff M. E. (Malden, MA: WileyBlackwell).

[B10] Burgoon J. K. (2015b). When is deceptive message production more effortful than truth-telling? A baker's dozen of moderators. Front. Psychol. 6:1965. 10.3389/fpsyg.2015.01965 26733932 PMC4689870

[B11] Burgoon J. K. Kelley D. L. Newton D. A. Keeley-Dyreson M. P. (1989). The nature of arousal and nonverbal indices. Hum. Commun. Res. 16, 217–255. 10.1111/j.1468-2958.1989.tb00210.x

[B12] Burgoon J. K. Proudfoot J. G. Wilson D. Schuetzler R. (2014). Patterns of nonverbal behavior associated with truth and deception: illustrations from three experiments. J. Nonverbal Behav. 38, 325–354. 10.1007/s10919-014-0181-5

[B13] Caso L. Maricchiolo F. Bonaiuto M. Vrij A. Mann S. (2006). The impact of deception and suspicion on hand gestures. J. Nonverbal Behav. 30, 1–19. 10.1007/s10919-005-0001-z

[B14] Chovil N. (1991). Social determinants of facial displays. J. Nonverbal Behav. 15, 141–154. 10.1007/BF01672216

[B15] Cushing T. (2017). Confirmed: TSA's Behavioral Detection Program is Useless, Biased, and Based on Junk Science. Techdirt. Available online at: (www.techdirt.com/articles/20170209/10331936675/confirmed-tsas-behavioral-detection-program-is-useless-biased-based-junk-science.shtml)

[B16] DePaulo B. M. Lindsay J. J. Malone B. E. Muhlenbruck L. Charlton K. Cooper H. (2003). Cues to deception. Psychol. Bull. 129, 74–118. 10.1037/0033-2909.129.1.74 12555795

[B17] Driskell J. E. Salas E. Driskell T. (2012). Social indicators of deception. Hum. Factors 54, 577–588. 10.1177/0018720812446338 22908681

[B18] Duran N. D. Dale R. Kello C. T. Street C. N. Richardson D. C. (2013). Exploring the movement dynamics of deception. Front. Psychol. 4:140. 10.3389/fpsyg.2013.00140 23543852 PMC3608909

[B19] Ekman P. (2009). Telling Lies: Clues to Deceit in the Marketplace, Politics, and Marriage (Revised Edition). New York, NY: WW Norton.

[B20] Ekman P. Friesen W. V. (1969). Nonverbal leakage and clues to deception. Psychiatry 32, 88–105. 10.1080/00332747.1969.11023575 5779090

[B21] Fernández-Dols J.-M. Sanchez F. Carrera P. Ruiz-Belda M.-A. (1997). Are spontaneous expressions and emotions linked? An experimental test of coherence. J. Nonverbal Behav. 2, 163–177. 10.1023/A:1024917530100

[B22] Grammer K. Schiefenhövel W. Schleidt M. Lorenz B. Eibl-Eibesfeldt I. (1988). Patterns on the face: the eyebrow flash in crosscultural comparison. Ethology 77, 279–299. 10.1111/j.1439-0310.1988.tb00211.x

[B23] Grazioli S. Johnson P. E. Jamal K. (2006). A cognitive approach to fraud detection. J. Forensic Account. 7, 65–68. 10.2139/ssrn.920222

[B24] Hartwig M. Bond C. F. Jr. (2011). Why do lie-catchers fail? A lens model meta-analysis of human lie judgments. Psychol. Bull. 137, 643–659. 10.1037/a0023589 21707129

[B25] Hoque M. E. McDuff D. J. Picard R. W. (2012). Exploring temporal patterns in classifying frustrated and delighted smiles. IEEE Trans. Affect. Comput. 3, 323–334. 10.1109/T-AFFC.2012.11

[B26] Hurley C. M. Frank M. G. (2011). Executing facial control during deceptive situations. J. Nonverbal Behav. 35, 119–131. 10.1007/s10919-010-0102-1

[B27] Köhnken G. Milne R. Memon A. Bull R. (1999). The cognitive interview: a meta-analysis. Psychol. Crime Law 5, 3–27. 10.1080/10683169908414991

[B28] Le Poire B. A. Burgoon J. K. (1996). Usefulness of differentiating arousal responses within communication theories: orienting response or defensive arousal within theories of expectancy violation. Commun. Monogr. 63, 208–230. 10.1080/03637759609376390

[B29] Leal S. Vrij A. (2008). Blinking during and after lying. J. Nonverbal Behav. 32, 187–194. 10.1007/s10919-008-0051-0

[B30] Lee C.-C. Welker R. B. Odom M. D. (2009). Features of computer-mediated, text-based messages that support automatable, linguistics-based indicators for deception detection. J. Inform. Syst. 23, 5–24. 10.2308/jis.2009.23.1.24

[B31] Levine T. R. Blair J. P. Carpenter C. J. (2018). A critical look at meta-analytic evidence for the cognitive approach to lie detection: a re-examination of Vrij, Fisher, and Blank (2017). Legal Criminol. Psychol. 23, 7–19. 10.1111/lcrp.12115

[B32] Mullin D. S. (2012). Effects of Deceptive Behavior on Biomechanical Measures of Standing Posture. Unpublished master's thesis, University of Missouri-Kansas City.

[B33] Okubo M. Kobayashi A. Ishikawa K. (2012). A fake smile thwarts cheater detection. J. Nonverbal Behav. 36, 217–225. 10.1007/s10919-012-0134-9

[B34] Pentland S. J. Burgoon J. K. Twyman N. W. (2015). Face and head movement analysis using automated feature extraction software, in Proceedings of the 48th Annual Hawaii International Conference on System Sciences (Kauai).

[B35] Pentland S. J. Twyman N. W. Burgoon J. K. (2014). Automated analysis of guilt and deception from facial affect in a Concealed Information Test, in Society for Personality and Social Psychology (Austin, TX).

[B36] Pentland S. J. Twyman N. W. Burgoon J. K. Nunamaker J. F. Diller C. B. R. (2017). A video-based screening system for automated risk assessment using nuanced facial features. J. Manage. Inform. Syst. 34, 970–993. 10.1080/07421222.2017.1393304

[B37] Pentland S. J. Twyman N. W. Burgoon J. K. (2016). In search of reliable facial cues for deception detection, in Proceedings of the 49th Annual Hawaii International Conference on System Sciences (Kauai).

[B38] Pentland S. J. Zhang B. (2016). Identifying deception using facial motion capture and analysis, in Paper Presented at Workshop on Information Technology and Systems (WITS) (Dublin).

[B39] Porter S. ten Brinke L. (2008). Reading between the lies: identifying concealed and falsified emotions in universal facial expressions. Psychol. Sci. 19, 508–514. 10.1111/j.1467-9280.2008.02116.x 18466413

[B40] Porter S. ten Brinke L. (2010). The truth about lies: what works in detecting high-stakes deception? Legal Criminol. Psychol. 15, 57–75. 10.1348/135532509X433151

[B41] Porter S. ten Brinke L. Wallace B. (2012). Secrets and lies: involuntary leakage in deceptive facial expressions as a function of emotional intensity. J. Nonverbal Behav. 36, 23–37. 10.1007/s10919-011-0120-7

[B42] Sporer S. (2013). Bodily communication and deception, in Body – Language – Communication: An International Handbook On Multimodality in Human Interaction, eds Müller C. Cienki A. Fricke E. Ladewig S. H. McNeill D. Tessendorf S. (Berlin; Boston, MA: de Gruyter Mouton), 1913–1921.

[B43] Sporer S. (2016). Deception and cognitive load: expanding our horizon with a working memory model. Front. Psychol. 7:420. 10.3389/fpsyg.2016.00420 27092090 PMC4823263

[B44] ten Brinke L. Porter S. (2012). Cry me a river: identifying the behavioral consequences of extremely high-stakes interpersonal deception. Law Hum. Behav. 36, 469–477. 10.1037/h0093929 23205594

[B45] ten Brinke L. Porter S. Baker A. (2012). Darwin the detective: observable facial muscle contractions reveal emotional high-stakes lies. Evol. Hum. Behav. 33, 411–416. 10.1016/j.evolhumbehav.2011.12.003

[B46] Twyman N. W. Elkins A. C. Burgoon J. K. (2011). A rigidity detection system for the guilty knowledge test, in Proceedings of the 44th Annual Hawaii International Conference on System Sciences (CD-ROM) (Koloa: Computer Society Press).

[B47] Twyman N. W. Elkins A. Burgoon J. K. Nunamaker J. F. Jr. (2014). A rigidity detection system for automated credibility assessment. J. Manage. Inform. Syst. 31, 173–201. 10.2753/MIS0742-1222310108

[B48] Twyman N. W. Proudfoot J. G. Schuetzler R. M. Elkins A. C. Derrick D. C. (2015). Robustness of multiple indicators in automated screening systems for deception detection. J. Manage. Inform. Syst. 32, 215–245. 10.1080/07421222.2015.1138569

[B49] U.S. Government Accountability Office (2013). Aviation Security: TSA Should Limit Future Funding for Behavior Detection Activities. Testimony Before the Subcommittee on Transportation Security; Committee on Homeland Security; House of Representatives.

[B50] Vrij A. (2015). A cognitive approach to lie detection, in Deception Detection: Current Challenges and New Approaches, eds Granhag P. A. Vrij A. Verschuere B. (Chichester: Wiley), 205–229.

[B51] Warren G. Schertler E. Bull P. (2009). Detecting deception from emotional and unemotional cues. J. Nonverbal Behav. 33, 59–69. 10.1007/s10919-008-0057-7

[B52] Yan W. J. Wu Q. Liang J. Chen Y. H. Fu X. (2013). How fast are the leaked facial expressions: the duration of micro-expressions. J. Nonverbal Behav. 37, 217–230. 10.1007/s10919-013-0159-8

[B53] Zuckerman M. DePaulo B. M. Rosenthal R. (1981). Verbal and nonverbal communication of deception, in Advances in Experimental Social Psychology, Vol. 14, ed Berkowitz L. (New York, NY: Academic Press), 1–59.

